# Inhibition of Amphiphilic N-Alkyl-O-carboxymethyl Chitosan Derivatives on* Alternaria macrospora*

**DOI:** 10.1155/2018/5236324

**Published:** 2018-06-11

**Authors:** Qili Liu, Jianxin Zhang, Dong Li, Jianfeng Lang, Shasha Zai, Jianjun Hao, Xiaohui Wang

**Affiliations:** ^1^Postdoctoral Research Base, Henan Institute of Science and Technology, Xinxiang, China; ^2^College of Plant Protection, Henan Agricultural University, Zhengzhou, Henan, China; ^3^School of Food and Agriculture, The University of Maine, Orono, ME 04469, USA; ^4^College of Fisheries, Henan Normal University, Xinxiang, China; ^5^State Key Laboratory of Pulp & Paper Engineering, South China University of Technology, Guangzhou, China

## Abstract

Cotton leaf spot* (Alternaria macrospora)* is a widespread disease that occurs in the main cotton-producing area of China. In managing this disease, a novel chitosan-based biopesticide, an amphiphilic N-alkyl-O-carboxymethyl chitosan derivative, was prepared. The product was selected from variations of chitosan with different molecular structures, which were obtained via a two-step reaction. First, carboxymethyl chitosans with varying molecular sizes were obtained by etherification with chloroacetic acid; then the carboxymethyl chitosan was alkylated with C4–C12 fatty aldehyde through a Schiff-base reaction. This procedure resulted in derivatives of amphiphilic N-alkyl-O-carboxymethyl chitosan, which showed strong antifungal activities against* A. macrospora*, and the efficacy was determined by its molecular structure.

## 1. Introduction

Cotton leaf spot* (Alternaria macrospora)* is a widespread disease [1], which occurs in main cotton-producing area of China, particularly in the Northern area. The disease is more severe in damp and rainy years [1]. To control the disease, several strategies have been used, including chemicals. Some synthetic chemical fungicides have been effective for years until they were overcome by the pathogen. Their applications have also caused environmental pollution [2]. Thus, there is a need to develop novel strategies that are environment friendly for disease control [3].

Chitosan has been extensively studied due to its broad spectra of bioactivities, such as antimicrobial and antitumor and immune enhancing effects [4, 5]. In light of several biological properties including biodegradability, biocompatibility, antibacteria, and nontoxicity, chitosan can be used in waste water treatment, food processing, cosmetics, pharmaceuticals, biomaterials, and agriculture [5, 6]. Many studies have shown that the positive polyelectrolyte nature of chitosan can disrupt the completeness of bacterial cell membrane and inhibit the growth of fungi [4, 7–9]. Chitosan oligomers with various molecular weights have an inhibitory effect on* Bacillus megaterium*,* B. cereus*, and* Enterobacter sakazakii* [10]. EI Ghaouth reported that chitosan can inhibit the growth of* Alternaria alternata, Botrytis cinerea, Colletotrichum gloeosporioides*, and* Rhizopus stolonifera [9].* More importantly, chitosan can also stimulate natural defense response of a plant [11].

Chitosan is a weak cationic biopolymer obtained by partial deacetylation of chitin, which is the most abundant natural polysaccharide next to the cellulose and can be found in the skeletal materials of crustaceans and insects, and cell walls of bacteria and fungi [4, 9, 12]. Comparing to other synthetic chemical fungicides, chitosan is a natural polysaccharide with a chemical structure of poly *β*-(1,4) N-acetyl-D-glucosamine [13]. Chitosan's antimicrobial activities are thought to originate from its polycationic nature [14]. The antifungal activity is affected by cation, and quaternized chitosan has a better antifungal activity than chitosan, Schiff bases of chitosan, and N-substituted chitosan [15].

Although chitosan exhibits antimicrobial activity, it is only effective in acidic condition because it has poor solubility at pH above 6.5 and is insoluble in water and most organic solvents [4, 5]. Thus, it is expected to find water-soluble chitosan derivatives that are soluble to solutions with large range of pH values. From previous studies, we have learned that chemical modification of chitosan at the amino or hydroxyl sites at C-2, C-3, and C-6 positions of the glucosamine residue may improve its antimicrobial activities [16]; and carboxymethyl chitosan enhances antimicrobial activities due to the substitution of the hydroxyl group in chitosan with the acetyl groups, which enhances the protonation of the amine group in the C-2 position in the presence of the new carboxyl ion [17]. Moreover, since carboxymethyl chitosan (CCS) can be dissolved in water over a wide pH range, it has a much broader application potential as an antimicrobial agent than chitosan [11]. However, generally acetylation of chitin chitosan results in large molecular weight and a tight crystal structure, which is insoluble in common solvents but only dissolved in acidic medium. This greatly restricts the use of chitosan. In addition, molecular weight significantly affects the function of chitosan, with controversial characteristics [10, 18].

The goal of this study was to determine and characterize their antifungal activities of the derivatives of amphiphilic alkyl carboxymethyl chitosan (ACCS). The application of these products may contribute to improving disease management using environmentally friendly chemicals.

## 2. Materials and Methods

### 2.1. Chemicals and Fungal Culture

Chitosan (MW ≈ 5.0 × 10^5^ Dalton) with a degree of deacetylation of approximately 90% (Zhejiang Golden-Shell Biological Chemical Co. Ltd., Taizhou, China) was used after purification by 1% (w/v) acetic acid solution. Chloroacetic acid was purchased from Tianjin Good Morning Fuchen Chemical Reagent Factory (Tianjin, China). Lauric aldehyde (98% active ingredient or a.i.), decyl aldehyde (98% a.i.), *n*-caprylic aldehyde (98% a.i.), and *n*-butyl aldehyde (98% a.i.) were purchased from Shanghai Crystal Pure Reagent Co., Ltd. (Shanghai, China). Sodium borohydride was provided by the Chinese Medicine Group Chemical Reagent Co., Ltd. (Shanghai, China). Other reagents were commercially available analytical reagents or laboratory grade materials.

*Alternaria macrospora* was obtained from the Culture Collection Center of the Department of Plant Protection of Henan Institute of Science and Technology and incubated on potato dextrose agar (PDA) plates at 28°C. To induce spore production,* A. macrospora* culture was incubated under black-light lamp when the mycelial colony grew near the edge of medium plates. Fungal spores were obtained by flooding the culture with sterile distilled water containing 0.05% (v/v) Tween-80. Spore suspensions were filtered through four layers of sterilized cheesecloth to remove adhering mycelia. The spore concentration was adjusted to 1.0 × 10^7^ cfu/mL with the aid of a hemocytometer prior to use.

### 2.2. Synthesis of Amphiphilic N-Alkyl-O-carboxymethyl Chitosan

#### 2.2.1. Degradation of Chitosan

Chitosan (15 g) was dissolved in 2% glacial acetic acid solution, and 3% H_2_O_2_ was dropwise added, followed by incubation at 60°C for 0, 0.5, 1, and 2 h, respectively. The reaction was terminated with NaOH solution and the pH value of the solution was adjusted to 7. Precipitation was done by adding a large amount of ethanol to wash the samples by suction filtering. The prepared samples were freeze-dried and labeled as CS0, CS1, CS2, and CS3. The molecular weight of the samples was measured using water-soluble gel permeation column PL aquagel-OH 40 (Agilent, Palo Alto, USA). The sample was dissolved in a sodium acetate buffer (pH = 4.8) at a concentration of 0.1%.

#### 2.2.2. Synthesis of Carboxymethyl Chitosan (CCS)

**Figure 1:**
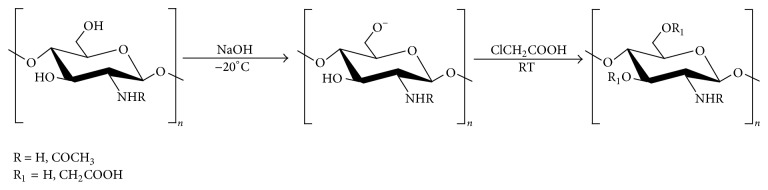
Synthetic route for carboxymethyl chitosan (CCS).

Five grams of degraded chitosan (CS0, CS1, CS2, and CS3) was added to 50% NaOH in a flask, stirred with a glass rod, and placed in a freezer overnight. The solution was transferred into a 1000 ml, three-necked flask, followed by adding with 10 ml of isopropanol. Five grams of chloroacetic acid was added in the flask and incubated for 12 h on a shaker. At this point, isopropanol was filtered off. The sample was dissolved in water. The solution pH of the sample was adjusted to 7.0 with dilute acetic acid, then transferred to a dialysis bag (MWCO = 3500) to dialyze for 3 to 5 days, and then freeze-dried to obtain dry CCS products (Figure 1).

CCS products with different molecular weights were prepared by the above method using chitosan having different molecular weights (CS0, CS1, CS2, and CS3) as raw materials. The carboxymethyl degree of substitution was measured by potentiometric titration method [10]. Specifically, 0.1 g CCS was dissolved in 30 ml 0.1 mol/L hydrochloric acid solutions, which were titrated with 0.1 mol/L NaOH solution. For every drop of 0.5 ml, pH values of abscissa consumption of NaOH volume, pH value of the vertical coordinate mapping, and calculation of CCS carboxymethyl degree of substitution were recorded.

#### 2.2.3. Calculation

Conductivity meter reading (DS) was used as the vertical axis, the consumption of sodium hydroxide (*V*) through the first-order and second-order differential graph inflection point. The degree of substitution (DS) is calculated as

(1) $$\begin{array}{c} DS=\frac{0.203x}{\left(1-0.059x\right)}, \end{array}$$

where *x* = (*V*2 − *V*1)*C*/*M*, *M* is CCS mass (g); *C* is for the use of sodium hydroxide in the molar concentration (mol/L); *V*1 is excess hydrochloric acid for the titration endpoint volume (m L); *V*2 of –COOH is for the titration of excess endpoint volume (m L), and 0.059\0.203 is for the reaction with 1 mol NaOH corresponding to the number of milligrams of acetyl glucosamine for the corresponding reaction with 1 mol NaOH CCS milligrams [19].

#### 2.2.4. Synthesis of Alkyl Carboxymethyl Chitosan (ACCS)

**Figure 2:**
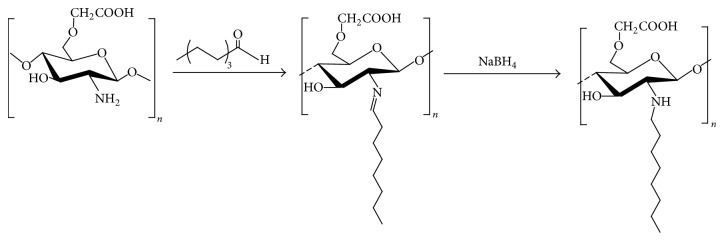
Synthetic route for alkyl carboxymethyl chitosan (ACCS).

The above CCS products were dissolved in 50 ml 0.2 M acetic acid solution, followed by adding 10 ml anhydrous ethanol with stirring. The ethanol solution of aliphatic aldehyde with different chain length (C4, C8, C10, and C12) was mixed with CCS solution at a molar radio of 2 : 1 to initiate a Schiff-base reaction for 4 h. An aliquot of 10 ml NaBH_4_ was added to the reaction by stirring for 12 h. The mixed solution was transferred to a dialysis bag [molecular weight cutoff (MWCO) = 3500] to dialyze for 3 to 5 days, followed by freeze drying. Alkylated carboxymethyl chitosan (ACCS) was then collected (Figure 2).

#### 2.2.5. Instrumental Characterization

Bruker TENSOR 27 type Fourier transform infrared (FTIR) spectrometer was used to measure infrared spectra of CCS and ACCS. Samples (2–5 mg) and 0.1 g potassium bromide were mixed and grinded and scanned at 4000 to 400 each centimeter. NMR spectrum was measured using a Bruker 400 MHz NMR and CD_3_COOD was chose as solvent. Tetramethylsilane (TMS) was an internal standard and the sample concentration was 10 mg/ml. X-ray diffraction spectra were detected by the XD-3A-ray diffractometer, Cu target, and K*α* ray. The acceleration voltage was 40 kV and accelerating current was 30 mA. The scan range 2*θ* = 6 to 60°. Thermogravimetric spectrum diagram was measured by Q500 TGA thermogravimetric analyzer (TA Instruments, USA). Temperature range was set from 30 to 600°C and heating rate was 10°C per minute.

### 2.3. Antifungal Activities Test

Antifungal activity of chitosan derivatives was assayed on an agar plate [9]. A culture plug (5 mm in diameter) of* A. macrospora* was transferred to a Petri plate containing potato dextrose agar (PDA) amended with one of the prepared chemicals. PDA plates without adding chemicals were used for control. Mycelial growth rate was calculated by measuring the size of colony.

#### 2.3.1. Chemical Preparation

Acetic acid (1%) solution was used to dissolve the chemicals to 5 mg/mL as a stock solution, which then further diluted to serial concentrations: 1, 0.5, 0.25, 0.1, 0.05, 0.025, 0.01, 0.005, and 0.001 mg/mL. To investigate the effect of molecular weights on the antifungal activities of amphiphilic chitosan derivatives, raw chitosan was degraded in H_2_O_2_ for a different time to obtain degraded chitosan with molecular weights varying in the range of 8.2 to 44 kDa.

#### 2.3.2. Effective Concentration for 50% Inhibition (EC50)

One milliliter chitosan solutions at serial concentrations were transferred onto a PDA plate when the medium (60°C, 12 mL) was poured, followed by slight mixing. This resulted in PDA plates amended with various concentrations of chitosan. PDA mixed with 1% dilute acetic acid, sterile water, and chlorothalonil (analytical pure) were used for controls. Agar disk (8 mm in diameter) of* A. macrospora* was transferred on the center of one of the prepared agar plates. Each treatment was repeated 3 times. After incubation at 28°C for 24, 48, and 72 h, respectively, the colony radius was measured. Inhibition rate of mycelial growth was calculated based on the colony size. EC50 values of chemicals were calculated according to the mycelium growth rate [7].

#### 2.3.3. Effect of Chitosan on the Morphology of* A. macrospora*

After culturing at 28°C for 72 h, the fungal colony and sporulation of* A. macrospora* were observed visually or under a microscope. Mycelia were cut from the edge of the colony and transferred to a microscopic slide for observation. A small amount of methylene blue was added to the mycelia and kept 5 to 8 min. After drying with a flame of alcohol burner, the form of mycelium and the spores were observed using an optical microscope.

#### 2.3.4. Statistical Analysis

All statistical analyses were performed with SPSS 11.5 (SPSS Inc., Chicago, IL, USA). Data were analyzed by one-way ANOVA. Mean separation was performed by Duncan's multiple range tests with significance level *α* = 0.05.

## 3. Results and Discussions

### 3.1. Production of Carboxymethyl Chitosan (CCS) and Amphiphilic Alkyl Carboxymethyl Chitosan (ACCS)

**Figure 3:**
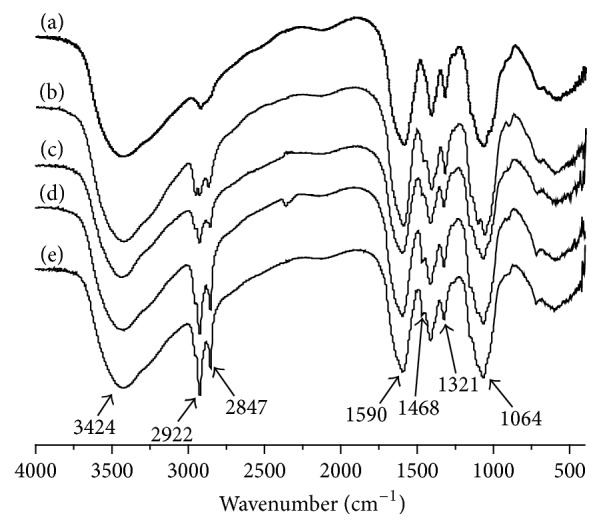
Fourier transform infrared spectra of carboxymethyl chitosan (CCS) and four alkyl carboxymethyl chitosan (ACCS) with different chain length: (a) CCS, (b) C4-CCS, (c) C8-CCS, (d) C10-CCS, and (e) C12-CCS.

The carboxymethyl derivatives of the chitosan samples with various molecular weights were synthesized by etherification with chloroacetic acid under mild basic condition (Figure 3) FTIR spectra of the CCS sample, in which CCS had absorptions at 1590 cm^−1^ and at 1064 cm^−1^ indicating the resulting synthesis of O-CMC. The degree of carboxymethyl substitution was determined by potentiometric titration (Table 1).

With the extended degradation time, the yield showed a gradual increase. When the yield reached a certain value, it began a decreasing process. The highest yield was 99.3% when the degradation time was 1.5 h. The molecular weight was continuously decreased with the increase of degradation time. In this case, the initial molecular weight is 12,000 kDa. After 2 h degradation time, the molecular weight was 8.2 kDa. Different molecular weights of CCS had different yields. When the substitution of carboxymethyl degree was 0.84, the yield was the highest (72.5%) and the molecular weight was 73.4 kDa. When the substitution of the highest carboxymethyl degree was 1.43, the yield only has 48.4%, and the molecular weight was 44.6 kDa. In all, while the chitosan degradation time is 2 hours, we could obtain the CCS molecular weight to a minimum, and its yield was relatively high as 56.4%.

FTIR spectra of CCS and modified with alkyl ester with varying carbon chain length are displayed in Figure 3. In comparison with CCS in spectrogram (a), the FTIR spectrum of alkylation presented stronger absorption peaks at 2922 cm^−1^ and 2847 cm^−1^, which may be attributed to the *υ*_CH3_ and *υ*_CH2_ stretching bands of ACCS. Meanwhile, the band appearing at 1468 cm^−1^ is new absorption peak of in-plane bending vibration (–CH_3_– and –CH_2_–). The above phenomenon proved the occurrence of alkylation reaction. Simultaneously, the FTIR spectrum also indicates that proportion existed between the absorption intensity of modified alkyl peak and the grafted alkyl chain length. These indicates that alkyl ester with varying carbon chain length have been grafted on the chitosan chain successfully.

### 3.2. NMR Analysis

**Figure 4:**
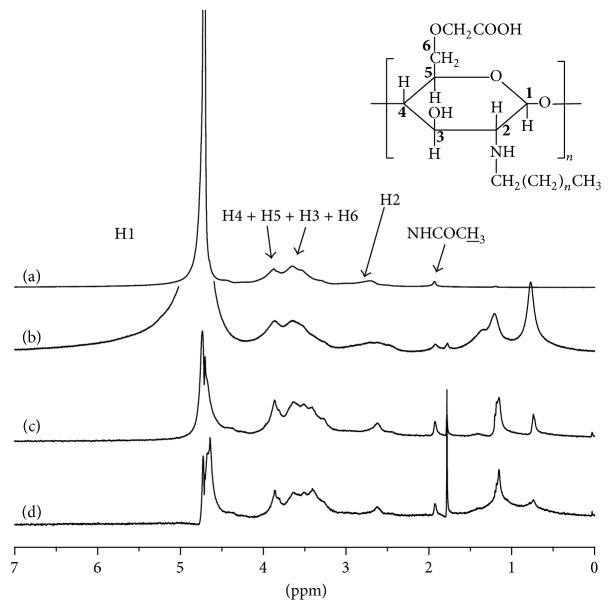
^1^H NMR spectra of carboxymethyl chitosan (CCS) and alkyl carboxymethyl chitosan (ACCS) with different chain length: (a) CCS, (b) C_4_-CCS, (c) C_10_-CCS, and (d) C_12_-CCS.

New signals at *δ* = 1.9 ppm and *δ* = 2.7 ppm corresponding to the methyl (acetyl) and H2 were observed (Figure 4(a)). The signals ranging from 3.5 ppm to 3.9 ppm were ascribed to the H3, H4, H5, and H6 protons of the main chain in chitosan, respectively. In contrast, the signals at *δ* = 0.7 ppm and *δ* = 1.7 ppm correspond to the characteristic peaks of methyl and methylene. This indicated that the alkyl chain was substituted on the CCS, which is consistent with infrared results (Figures 4(b), 4(c), and 4(d)).

### 3.3. X-Ray Analysis

**Figure 5:**
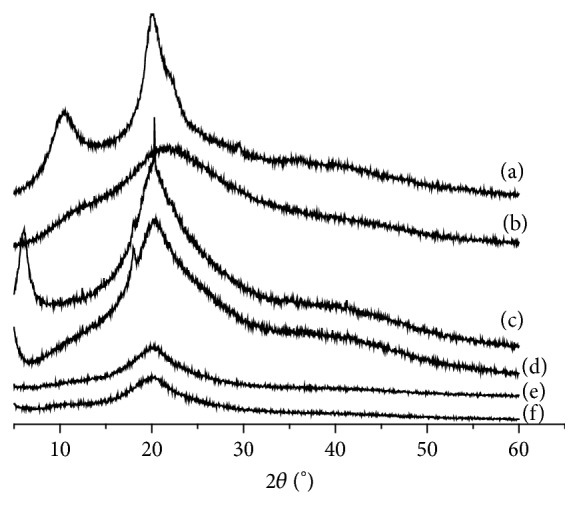
XRD spectra of chitosan (CS) and its derivatives with different chain length: (a) CS; (b) CCS; (c) C_4_-CCS; (d) C_8_-CCS; (e) C_10_-CCS; (f) C_12_-CCS.

Three diffraction peaks at 2*θ* of 10, 20, and 22° indicated that chitosan had a higher crystallinity degree (Figure 5). Only one smaller peak was observed in the diffraction pattern at 2*θ* = 20° of chitosan derivative after carboxymethyl modified, suggesting that original crystalline structure of chitosan was decreased by the introduction of carboxymethyl side chains. On the other hand, a new peak at 2*θ* = 20° was enhanced for alkylation modified CCS. This indicated that the hydrogen bonds between the molecules of the chitosan derivative were formed again so that the degree of crystallinity has increased due to the introduction of the alkyl group, which decreased again with the gradual increase of the substituted alkyl chain, suggesting the ordered structure of the original molecule was destructed (Figure 5).

### 3.4. Thermal T Analysis

**Figure 6:**
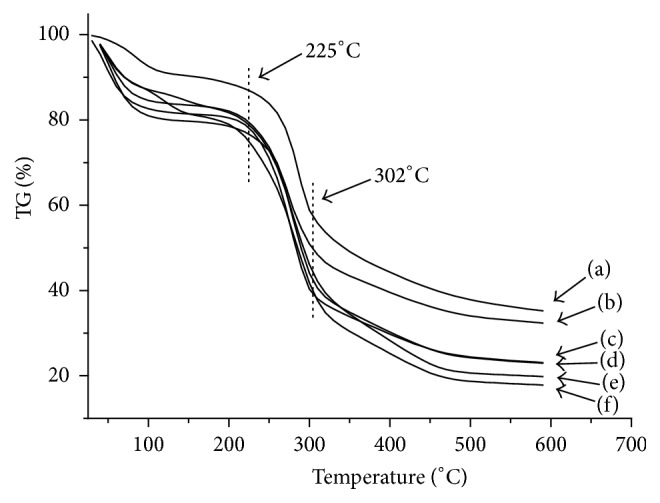
TG of chitosan (CS), carboxymethyl chitosan (CCS), and its derivatives with different chain length: (a) CS; (b) CCS; (c) ACCS1; (d) ACCS2; (e) ACCS3; (f) ACCS4.

There was a significant weight loss from 225 to 302°C in chitosan due to the degradation of chitosan chain. However, at the same temperature, the weight loss of chitosan increased with the increase of alkylated chitosan substituted group chain length. This indicated that, with the introduction of an alkyl group, the thermal stability of chitosan derivative decreased (Figure 6).

### 3.5. Antifungal Activities

**Figure 7:**
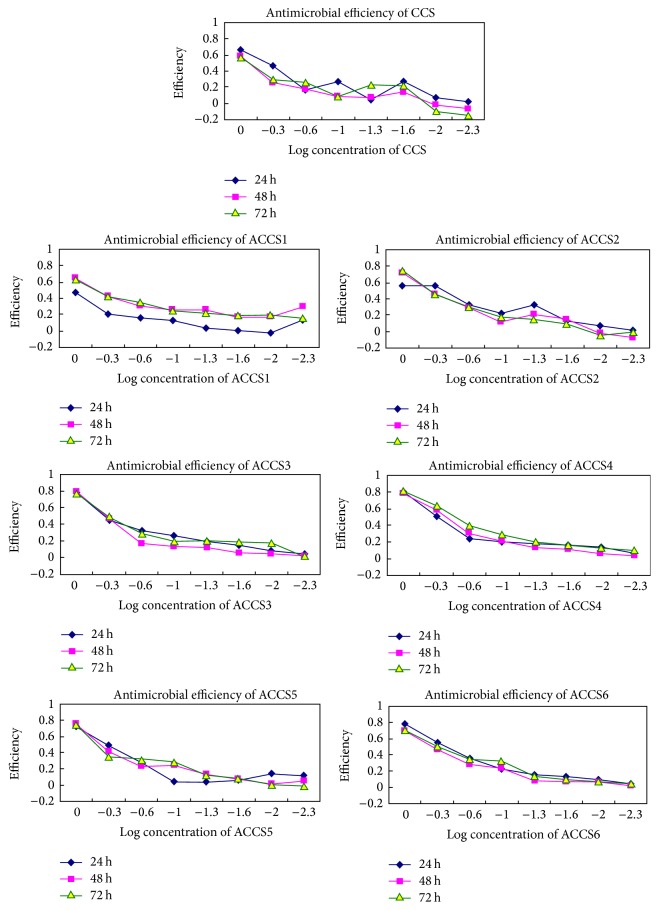
Antifungal effects of different compounds at 1 mg/mL concentration after different time of incubation. CCS: carboxymethyl chitosan; ACCS1–ACCS6: different proportions of modified CCS.

All the drugs had inhibitory effects on the fungus, with efficacies varied (Figure 7). Among CCS and its derivatives with different chain length, ACCS4 (C12) had the highest antifungal effects. EC50 of ACCS1 were significantly higher than other type chemicals and its side chain length was C4 (Table 2). The orders of EC50 value were as follows: from high to low: ACCS1 (C4), CCS (0), ACCS2 (C8), ACCS5 (C8), ACCS3 (C10), ACCS6 (C8), ACCS4 (C12), and chlorothalonil. But ACCS3 (C10) and ACCS4 (C12) had no significant difference with ACCS6 (C8) and chlorothalonil, respectively.

**Figure 8:**
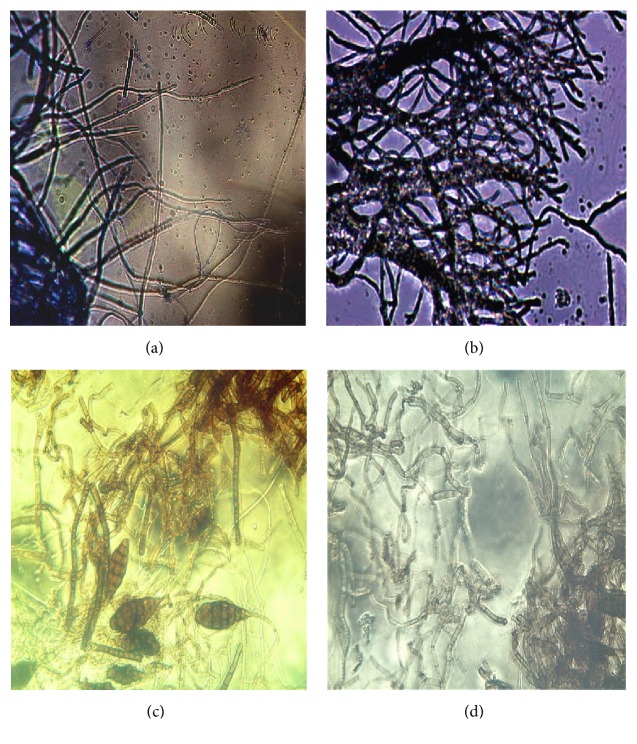
Effects of chemical treatment on morphologies of* Alternaria macrospora *after culturing for 3 days. (a) Mycelia on drug-free medium (no conidia were found). (b) Mycelia on ACCS4-amended medium. (c) Conidia and on ACCS4-amended culture medium. (d) Mycelia on chlorothalonil-amended medium.

Typically, hyphal cells of nontreated culture of* A. macrospora* were slender; the tip of hyphae was sharp and all the hyphae presented expanding state (Figure 8(a)). When incubated on ACCS4-amended medium, the hyphal development was significantly inhibited. The cytoplasm of hyphae concentrated, and the distance of neighboring diaphragms was significantly shorter (Figure 8(b)). The hyphae became thick and their tips were blunt and round (Figure 8(c)). The time of spores appearing in drug-amended medium was 2–5 d earlier than the negative control. The mycelia growing on chlorothalonil-amended medium had similar performance as on ACCS4-amended medium (Figure 8(d)).

The efficacy of chitosan's antimicrobial activity was influenced in the following four categories: (1) microbial factors, such as species and cell age; (2) intrinsic factors of chitosan, including positive charge density, molecular weight, concentration, hydrophilic/hydrophobic characteristics, and chelating capacity; (3) physical state, namely, water solubility and solid state of chitosan; and (4) environmental factors, involving ionic strength of medium, pH, temperature, and reactive time [11]. In this study, derivatives of amphiphilic N-alkyl-O-carboxymethyl chitosan with different chain lengths inhibited the growth of* A. macrospora *to a variable extent.* Alternaria macrospora* is a pathogen which causes diseases on* Anemone vitifolia *Buch. [1],* Solanum lycopersicum* [20], and* Parthenium hysterophorus* [21]. All ACCS derivatives exhibited much higher levels of antifungal activities compared with the original chitosan. The antifungal activity was enhanced with the increasing of chain length in this study. This result may be due to two reasons. One reason was that the amphiphilic chitosan derivatives led to easier combination with the target site of fungi. The second reason was that the chitosan derivatives with longer chain length had higher molecular weights which can display greater activity than low-molecular-weight chitosan. Although the efficacy of antifungal activity of ACCS4 (C12) did not significantly outcompete synthetic pesticides, it has a potential of application, as it does not cause any environmental pollution. It can be anticipated that, in the further research, more and more products will be derived by modifying the structures of natural compounds to enhance antifungal activities of these products.

## 4. Conclusions 

Novel chitosan-based biopesticides with different molecular structures can be synthesized by a two-step reaction: first, CCS with varying molecular size was obtained by etherification with chloroacetic acid; then the CCS was alkylated with C4–C12 fatty aldehyde through a Schiff-base reaction. The novel amphiphilic N-alkyl-O-carboxymethyl chitosan derivatives showed significant antifungal activities against* A. macrospora* and their efficacy was affected by molecular structure.

## Figures and Tables

**Table 1:** Synthesis of carboxymethyl chitosan (CCS) with different molecular weights (MW).

Chitosan	Degradation time (h)	Yield (%)	MW (kDa)	CCS	Derivatives	Yield (%)	MW (kDa)
CS0	0	-	50.0	CCS0	0.84	72.5	73.4
CS1	0.5	94.6	44.0	CCS1	1.43	48.4	44.6
CS2	1	98.6	27.0	CCS2	1.16	35.6	26.7
CS3	2	96.6	8.2	CCS3	1.14	56.4	12.1

*Note*. CS0–CS3: chitosan (CS) products with different degradation times; CCS0–CCS3: derivatives of CCS; MW: molecular weight.

**Table 2:** Effective concentration for 50% inhibition (EC50) to *Alternariamacrospora* of test chemicals and their characteristics.

Compound (starting material)	Side chain length	EC_50_ mg/ml
CCS (CCS 1)	0	0.392^ab^
ACCS 1 (CCS 1)	C4	0.422^a^
ACCS 2 (CCS 1)	C8	0.243^b^
ACCS 3 (CCS 1)	C10	0.212^b^
ACCS 4 (CCS 1)	C12	0.169^c^
ACCS 5 (CCS 2)	C8	0.236^b^
ACCS 6 (CCS 3)	C8	0.196^bc^
Chlorothalonil (−)	-	0.159^c^

Mean values followed by different letters are significantly different (*P* < 0.05). CCS: carboxymethyl chitosan; ACCS: alkyl carboxymethyl chitosan.
